# Hemosuccus Pancreaticus: A Serious Complication of Chronic Pancreatitis

**DOI:** 10.7759/cureus.25970

**Published:** 2022-06-15

**Authors:** Dayana Nasr, Abdul Bhutta, Pujitha Kudaravalli, Alyssa Ionno, Ganesh Aswath

**Affiliations:** 1 Internal Medicine, State University of New York (SUNY) Upstate University Medical Hospital, Syracuse, USA; 2 Gastroenterology, State University of New York (SUNY) Upstate University Medical Hospital, Syracuse, USA; 3 Radiology, State University of New York (SUNY) Upstate University Medical Hospital, Syracuse, USA

**Keywords:** hematochezia, hemorrhage, pseudocyst, chronic pancreatitis, hemosuccus pancreaticus

## Abstract

Hemosuccus pancreaticus is a rare cause of gastrointestinal bleeding that usually presents with melena and abdominal pain. It is defined as a hemorrhage from the ampulla of Vater passing through the main pancreatic duct toward the second portion of the duodenum. Imaging is usually required to establish a diagnosis, and angiography continues to be the gold standard for both treatment and diagnosis. In some instances where bleeding is uncontrolled or if the patient is unstable, surgery may be required. Physicians should have a high index of suspicion, especially in patients with a history of chronic pancreatitis, as this diagnosis is associated with a very high mortality rate if left untreated. We report a case of a 67-year-old male with a known history of chronic pancreatitis and pancreatic pseudocyst who presented with melena and right upper quadrant abdominal pain and was found to have hemosuccus pancreaticus secondary to a gastroduodenal artery bleed. He underwent successful angiographic embolization and was discharged home after ensuring resolution of bleed and improvement in symptoms.

## Introduction

Hemosuccus pancreaticus (HP) is described as a hemorrhage from the ampulla of Vater passing through the main pancreatic duct toward the second portion of the duodenum. It is an extremely rare cause of gastrointestinal (GI) bleed. The intensity of the bleed can vary from an occult GI bleed to a life-threatening hemorrhage. It was first described as early as 1931 and has been assigned different nomenclatures, including wirsungorrhaghia and pseudohemobilia [1]. Seeing the scarcity of cases reported, it remains an unfamiliar and challenging diagnosis to physicians. We herein describe the case of a patient who presented with a GI bleed and was found to have HP.

## Case presentation

**Figure 1:**
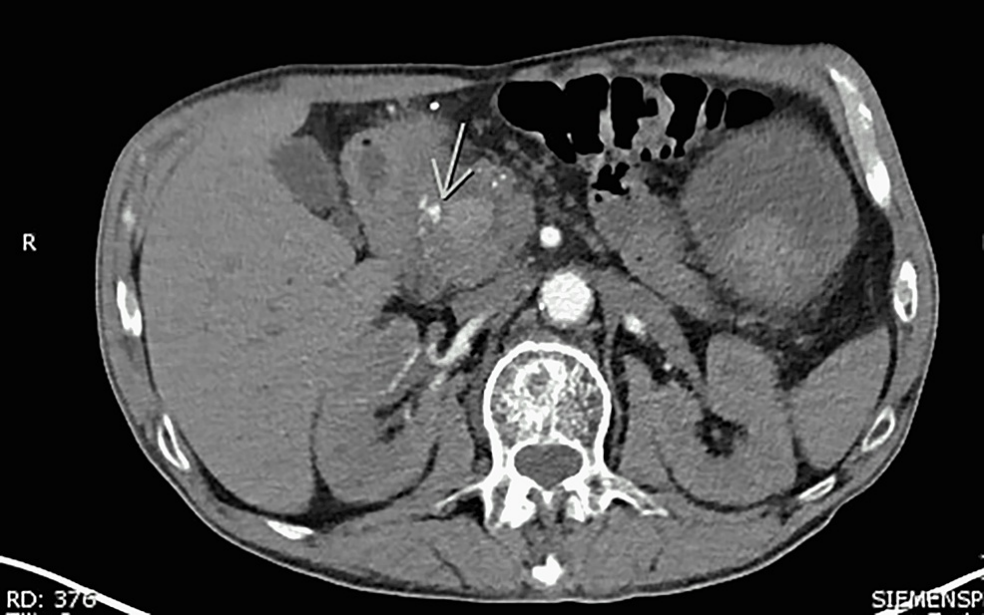
CT angiography of the abdomen and pelvis Axial CTA image of the abdomen and pelvis showing active extravasation into pancreatic pseudocyst (green arrow).

A 67-year-old male with a past medical history of chronic pancreatitis secondary to alcohol use, with a large, known pseudocyst measuring around 7.5 cm near the head of the pancreas, was transferred to our hospital for suspected pseudocyst bleed. He initially presented for right upper quadrant abdominal pain and melenic stools. He was hemodynamically stable, and physical examination showed mild epigastric tenderness and dark stools on rectal exam. Labs were significant for a hemoglobin of 6.3 g/dl, hematocrit of 19.9 %, lactate of 7.1 mmol/l, and elevated blood urea nitrogen (BUN) to creatinine ratio. A computed tomography (CT) scan of the abdomen with contrast revealed stable cyst size but possible pseudoaneurysm bleed via superior mesenteric artery (SMA) branches. This was followed by CT angiography (CTA), which ruled out active SMA bleed but did show abnormal vascular structures from the superior mesenteric vein (SMV) adjacent to the pseudoaneurysm. In addition, the CT scan was concerning for hemorrhage in the anterior aspect of the pseudocyst (Figure 1). Resuscitation with IV fluids and blood products was initiated.

**Figure 2:**
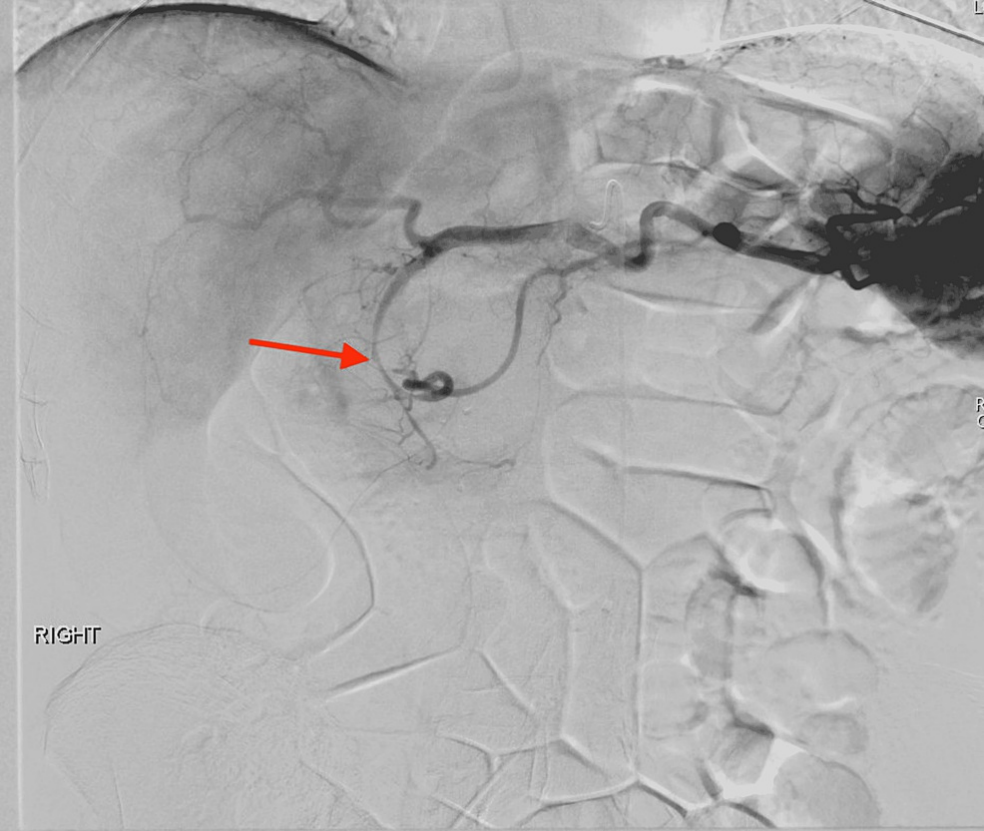
Celiac arteriogram Celiac arteriogram demonstrating an irregular appearing GDA with possible subtle pseudoaneurysm in its midportion (green arrow). GDA: gastroduodenal artery

**Figure 3:**
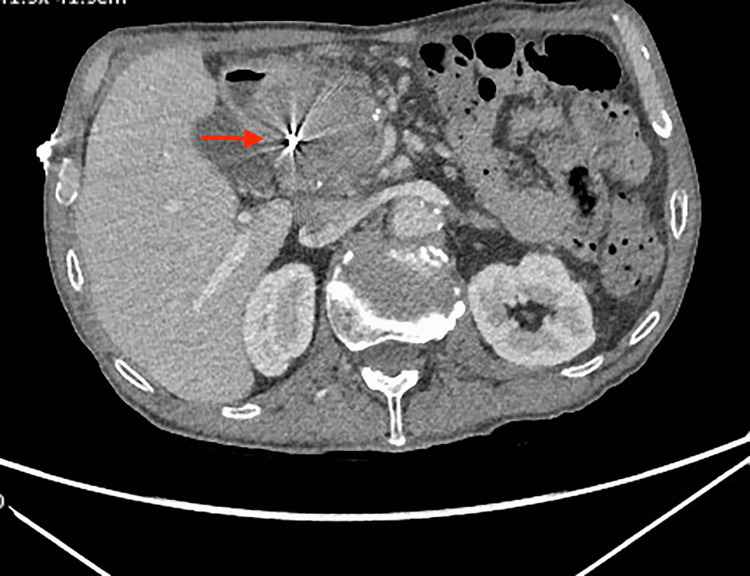
CT abdomen pelvis with contrast Axial CT image showing post-embolization coils (red arrow)

Gastroenterology and surgery recommended potential angioembolization by interventional radiology (IR). IR performed a visceral arteriogram that showed a diseased and irregular gastroduodenal artery (GDA) (Figure 2). The patient then successfully underwent embolization of GDA and its two main distal branches with multiple coils and gelfoam (Figure 3).

A repeat CTA was performed four days post-embolization and showed evidence of a hemorrhagic pseudocyst with no active extravasation. Surgery was deferred at this time.

The patient remained clinically and hemodynamically stable after the embolization with no further drop in hemoglobin. He was discharged with an outpatient hepatobiliary service follow-up. A repeat MRI three weeks later showed a decrease in the size of the pseudocyst and a repeat complete blood count (CBC) showed improved hemoglobin compared to discharge labs.

## Discussion

Hemosuccus pancreaticus is defined as bleeding from the ampulla of Vater via the pancreatic duct. The origin of the bleed can be from the duct, the pancreas, or even the structures around the pancreas including the splenic artery and the gastric artery [2]. According to a Medline search done by Tarar et al., only 123 cases have been reported so far [3].

Common entities that could be responsible for HP are chronic or hereditary pancreatitis, which is considered the most common cause. A pancreatic pseudocyst, present in around 10% to 17% of chronic pancreatitis cases, would further increase inflammation (thought to be secondary to an increase in elastase secretion) and cause lysis of the vessels’ wall, resulting in hemorrhage [4]. Finally, pancreatic tumors can also lead to the development of HP, but the pathophysiology remains unclear to date [2].

The most common presenting symptom is melena [5]. Abdominal pain is also common and can be explained by the rapid distention of the pancreatic duct by blood [6].

Due to the nonspecific features, patients usually undergo extensive workup before a diagnosis of HP is reached. Initial laboratory findings are nondiagnostic and might include elevated bilirubin due to pancreaticobiliary reflux [3]. Endoscopy mostly helps exclude other causes of GI bleeding.

Multiple imaging modalities exist that can aid in the diagnosis, including upper endoscopy, CT scan, magnetic resonance imaging, visceral angiography, endoscopic retrograde cholangiopancreatography (ERCP), endoscopic ultrasound, Doppler ultrasonography, and radionuclide scintigraphy.

Angiography is the gold standard procedure for both diagnosis and treatment [7]. Although it is associated with less morbidity and mortality than surgery, it has its limitation. In addition, some of the complications that may arise during an angiographic embolization include rupture of the pseudoaneurysm during embolization, rebleeding, splenic infarction, and coil migration [8].

In 17%-37% of the cases, surgical intervention might be required, especially if angiographic embolization fails or in the event of rebleeding [9].

## Conclusions

Hemosuccus pancreaticus is a rare but fatal diagnosis. HP should be included in the differential diagnosis when patients present with intermittent bleeding and abdominal pain. It is important to recognize this diagnosis given its associated morbidity and mortality. The choice of treatment should be on a case-by-case basis depending on the patient’s presentation and associated complications.
